# CIRCumcision learning experience using simulation: A pilot learning platform for safe neonatal circumcision training offered either virtually or in person

**DOI:** 10.3389/fruro.2023.1199194

**Published:** 2023-07-07

**Authors:** Joana Dos Santos, Abdulrahman Alsabban, Max Maizels, Michael Chua, Sunayna Vuppal, Emily Louca, Martin Perlmutar, Jennifer Knabl, Mandy Rickard, Abby Varghese, Armando J. Lorenzo, Martin Allan Koyle

**Affiliations:** ^1^ Division of Urology, Department of Surgery, The Hospital for Sick Children, Toronto, ON, Canada; ^2^ Department of Urology, Faculty of Medicine, King Abdulaziz University, Jeddah, Saudi Arabia; ^3^ Department of Urology, Northwestern University Feinberg School of Medicine, Chicago, IL, United States; ^4^ Institute of Urology, St. Luke’s Medical Center, Quezon City, Philippines; ^5^ Department of Pediatrics, Michael Garron Hospital, Toronto, ON, Canada

**Keywords:** neonatal circumcision, newborn, safe, education, circumcision training, virtual learning

## Abstract

**Background:**

To our knowledge, no formal training combining didactic learning, simulation, and hands-on performance is available for practitioners performing neonatal circumcision. The absence of structured training may result in avoidable complications such as bleeding and penile injury. Herein, we present the results of a pilot neonatal circumcision training platform, offered either virtually or in person.

**Material and methods:**

CIRCLES (CIRCumcision Learning Experience using Simulation) consist of 1. online didactic learning; 2. live simulation practice (in person or virtual coaching), and 3. clinical performance. Outcome measures included pre- and post-knowledge scores, self-efficacy questionnaires, and skill assessments of simulation and clinical performance (Likert rating). Face validity for training success was determined by an 80% passing score on the knowledge test and > 75% (mostly independent) performance.

**Results:**

For this pilot, we restricted enrolment to seven pediatric residents and one nurse practitioner. Wilcoxon Sum Rank test for non-parametric paired samples for pre-and post-knowledge tests showed a median increase of 20 points in post-knowledge tests (p=0.011). Upon completion of the simulation training, all participants (8/8) have chosen to perform circumcision with the GOMCO clamp. Both in-person (4/4) and virtual participants (4/4) performed >75% of simulation and clinical circumcision independently. Post-training self-efficacy Z scores were higher than pre-training scores, except for the management of bleeding.

**Conclusion:**

The pilot CIRCLES learning shows face validity for both in-person and virtual training for neonatal circumcision. We plan to extend this platform to include more trainees and to offer them to established practitioners. The availability of formal training may ultimately reduce adverse outcomes.

## 1 Introduction

Neonatal circumcision is one of the most commonly performed surgical procedures worldwide. It is estimated that 1.2 million newborn males are circumcised annually in the United States and 30% of newborn males in Canada are circumcised (1, 2). Despite the frequency of this procedure, no formal training is available to healthcare practitioners performing newborn circumcision (PPC), the vast majority of whom are non-surgeons and hence not procedurally based (pediatricians, family physicians, and allied health practitioners) [3,4,5]. PPC often learn from senior colleagues on the job, who invariably learn the procedure from other colleagues without circumcision-specific training (1). Although the risks associated with neonatal circumcision are often minor, rare but devastating complications, such as severe bleeding, penile injury, including glans and/or penile shaft amputation, and death, may be under-reported. Such complications likely occur because of the absence of structured training and may be preventable if performed by appropriately trained individuals (2–5).

We sought to create a pilot neonatal circumcision training platform consisting of: 1. online didactic learning; 2. simulation practice; and 3. hands-on clinical performance.

We hypothesized that the availability of an educational program for pediatric residents, including patients’ assessment of and performance of neonatal circumcision, would add value to their training and ultimately to the patients who they serve. The aim of our intervention was to develop and implement a combined didactic and hands-on circumcision simulation course offered to pediatric residents to improve their knowledge and confidence in performing neonatal circumcision, with the potential to ultimately reduce serious preventable complications.

Given the impact of COVID-19, we also wanted to compare the effectiveness of in-person versus virtual coaching in skills and knowledge gaining, and self-efficacy perception of participants in the circumcision simulation. Herein, we present the results of CIRCLES (CIRCumcision Learning Experience using Simulation) for neonatal circumcision.

## 2 Material and methods

We developed a 2-day pilot neonatal circumcision course, composed of didactic training and hands-on simulation training using Mogen and Gomco clamp techniques. After obtaining approval from our institutional Quality Improvement Committee, REB approval was waived. This course was offered to seven pediatric residents and one nurse practitioner, half of whom would participate in the simulation center and the others, virtually, using the Zoom platform.

### 2.1 CIRCLES task trainer development

**Figure 1:**
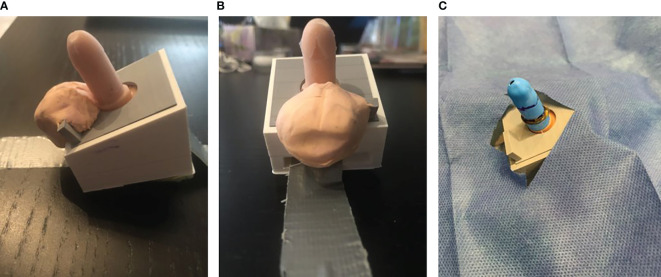
3D printed silicone task trainer with base attached to a table. **(A)** Lateral view demonstrating penopubic crease and penoscrotal junction (for the teaching of anatomical landmarks and areas of insertion of the needle for the ring penile block). **(B)** Ventral view demonstrating coronal edge and frenulum. An opening was designed horizontally through each base so that the task trainer could be secured to the table using tape. To provide greater stability, an arm was designed, 3D printed, and fixed to the bottom of the trainer to extend outward from the base. **(C)** 3D printed model with a balloon sleeve cut to the size of the silicone trainer. A hole was cut at one end to simulate the foreskin accurately.

The CIRCLES task trainer was created using the CAD software to design a mold for the initial model (**Figure 1**). This mold was then 3D printed and silicone was used to cast the object. A base and lid were designed, and 3D printed to house the silicone trainer and act as a body for the trainer. The silicone model fit within the base, the lid was secured over top and both 3D printed parts were fixed together using acetone. Balloons were cut to the size of the silicone trainer, and a hole was cut at one end to accurately simulate the foreskin. A piece of sponge was cut into a triangular shape and secured to the bottom of the task trainer in order to simulate the correct angle and movement of the model. An opening was designed horizontally through each base so that the task trainer could be secured to the table using a tape. To provide greater stability, an arm was designed, 3D printed and fixed to the bottom of the trainer to extend outward from the base. Clamps were purchased and used to hold the arm and the entire trainer to the table.

This 3D-printed, high-fidelity, reusable silicone mold of an infant male penis 3 cm in length was attached to a soft plastic base simulating the lower abdomen, thighs, and scrotum. The prepuce was simulated using a specifically designed latex sleeve placed over the penis.

**Figure 2:**
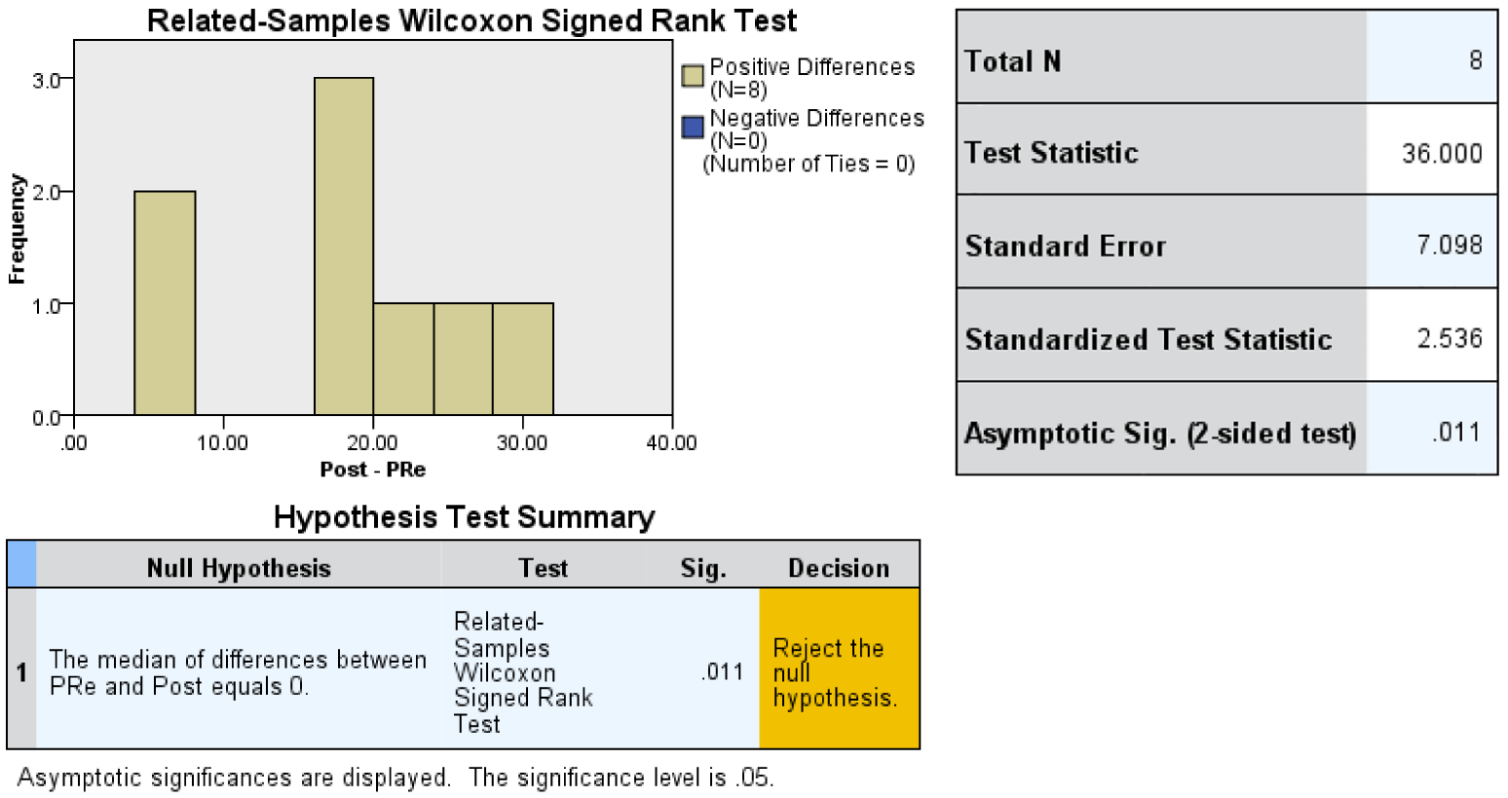
Wilcoxon Sum Rank test for pre- and post-knowledge test scores showing significantly higher scores post-training (p 0.011).

The instruments used for the simulated circumcisions were the same as those used in real circumcisions (**Figure 2**). Virtual participants received all of these instruments in addition to an HD webcam and a video link with installation and setup instructions one week prior to the course.

### 2.2 The simulation course, which we entitled CIRCLES included the following steps:

#### 2.2.1 Online didactic learning

##### 2.2.1.1 Pre-course accessible web-based comprehensive video learning module.

The module included videos of both simulated and clamp circumcisions in newborn males with a step-by-step checklist of the circumcision surgical technique. This included in-depth information regarding a) patient suitability, b) proper analgesia and equipment, c) clamp-specific technical aspects (Gomco and Mogen circumcision clamps), d) contraindications, and e) post-procedural care (6).

##### 2.2.1.2 Day 1 – morning session (virtual learning)

Upon completion of the web-based learning module, all participants were involved in this 3-hour online live learning session, which was interactive and based on pre-course materials.

#### 2.2.2 Simulation practice

##### 2.2.2.1 Day 1 – afternoon session (simulation lab)

**Figure 3:**
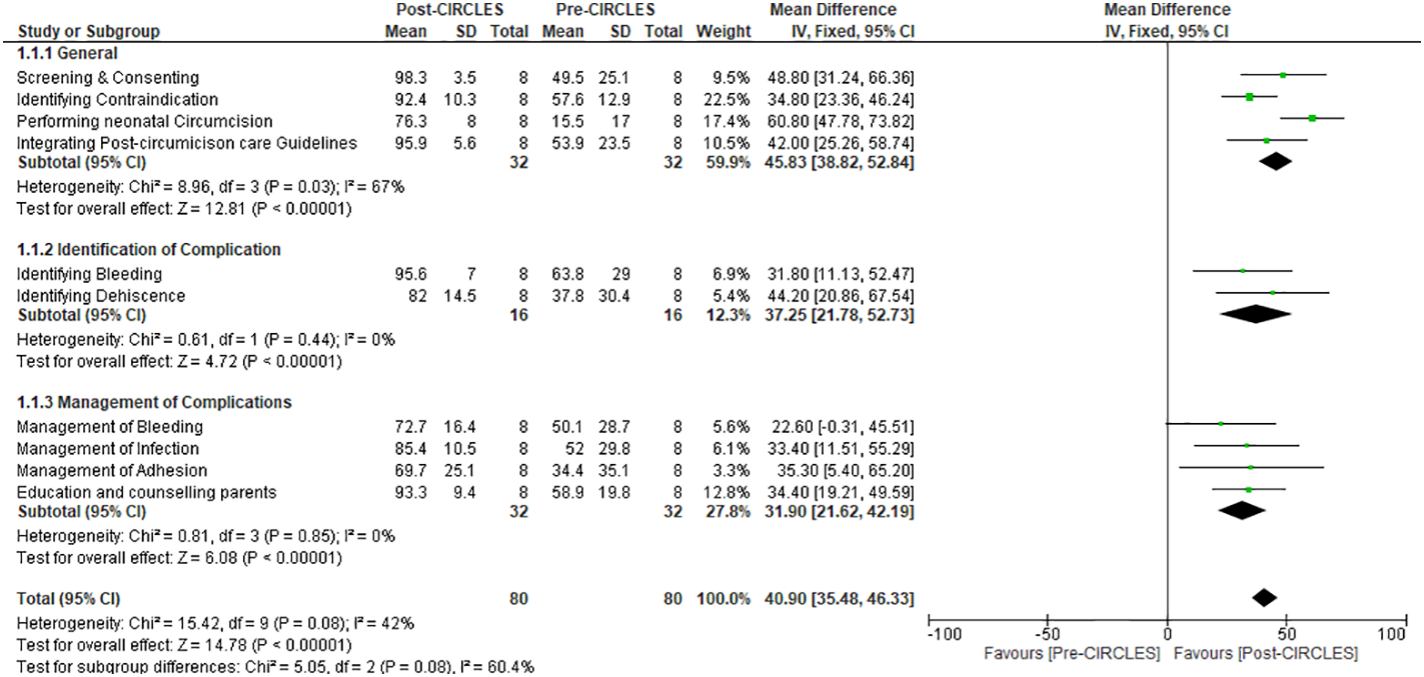
Forest plot chart comparing self-efficacy assessment of participants in all domains of confidence in neonatal circumcision pre- and post-CIRCLES.

**Figure 4:**
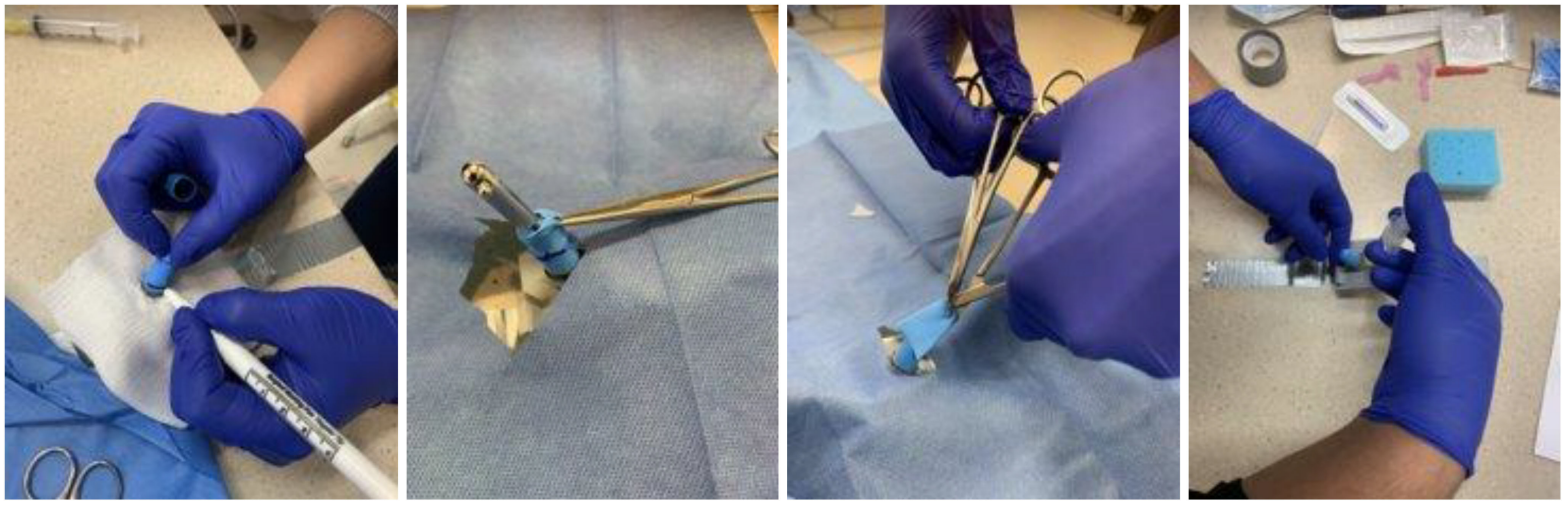
Simulation-Based Training In-Person (PRACTICE).

**Figure 5:**
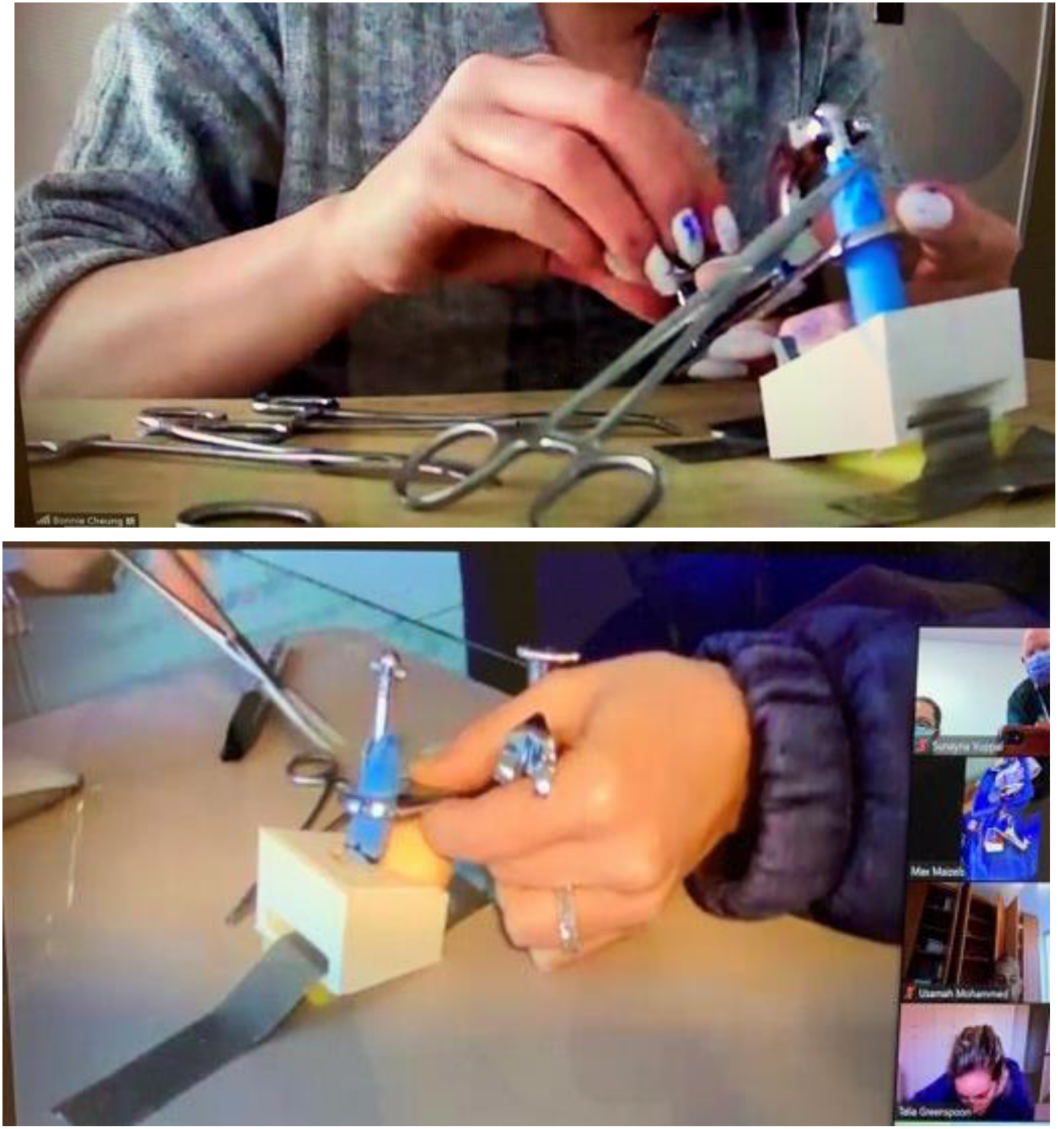
Simulation-Based Virtual Training (Practice).

This comprised a 3-hour hands-on simulation session. Facilitators demonstrated these techniques using a simulation task trainer. This included two in-person faculty facilitators and two virtual facilitators (on video, live), who demonstrated two circumcision modalities (Gomco and Mogen clamps) step-by-step, coaching and observing the participants’ progress as they practiced each technique (**Figures 3**–**5**).

##### 2.2.2.2 1-hour objective structured evaluation: At the end of simulation session

#### 2.2.3 Hands on clinical performance

##### 2.2.3.1 Day 2 (circumcision observation)

**Figure 6:**
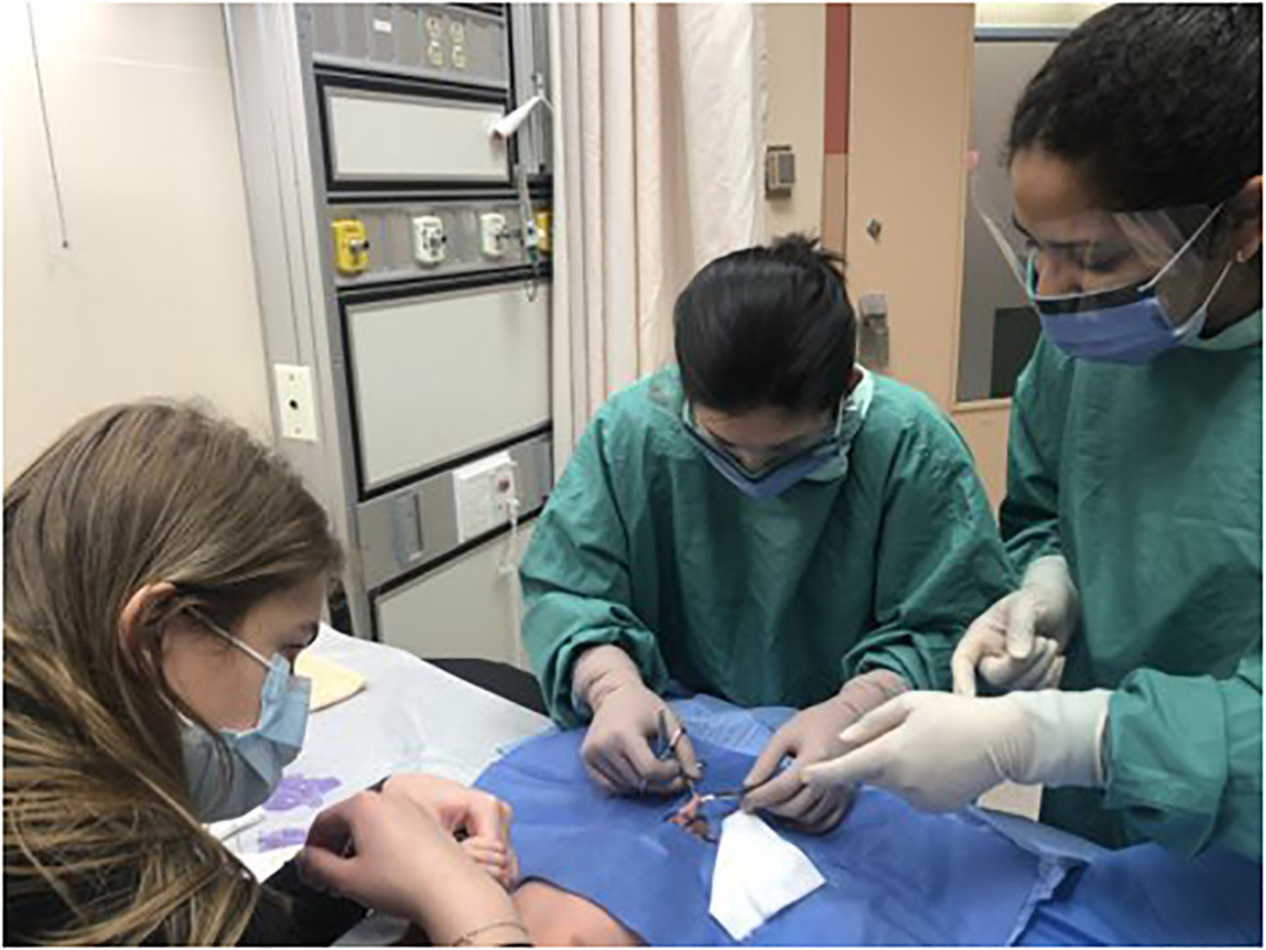
Clinical Performance with Urology Faculty Supervision (DO).

Direct observation and clinical performance of circumcisions at a tertiary pediatric hospital circumcision clinic under faculty supervision. A maximum of two participants at a time (**Figure 6**).

##### 2.2.3.2 Evaluations

Pre- and post-course knowledge tests and a detailed self-efficacy assessment were sent to participants and facilitators before day 1 and after day 2 of the course, respectively.

##### 2.2.3.3 Assessment of learning outcomes

Comparison of efficacy:1) learners’ pre and post-course skills, knowledge and self-efficacy scores; 2) comparison of learners’ pre and post-course skills, knowledge and self-efficacy scores who were assisted by in person facilitator and scores of learners assisted by virtual coach. Unidentified data and participants’ privacy were also protected.

The Wilcoxon Sum Rank test was used for non-parametric paired samples for the pre- and post knowledge tests.

For learners’ self-efficacy assessment pre- and post CIRCLES, continuous data of all domains of confidence in neonatal circumcision were analyzed and presented as mean change ± standard deviation from baseline, provided that the results were measured on the same scale. The confidence slider scale was rated from to 0-100, in which 0= cannot do at all, 50= moderately can do, and 100=highly certain can do so (**Supplemental Materials**). We conducted a z-score assessment for changes in mean values from baseline by applying the inverse variance method, fixed-effects model, and mean difference (changes from baseline) as the effect measure. We pooled data from simple parallel pre- and post-course questionnaires answered by all participants in CIRCLES. As the difference in means and the respective standard deviation were required for the analyses, we imputed those values for one missing participant. A subgroup analysis was performed to delineate competency in different phases of neonatal circumcision (**Figure 4**).

Face validity for training success was determined by showing an 80% passing score for knowledge and > 75% (mostly independent) performance of both simulation and clinical circumcision.

Additionally, learners answered experience surveys at the end of day 1 (didactic learning and simulation) and day 2 (clinical performance).

## 3 Results

Wilcoxon Sum Rank test showed a median increase of 20 points in participants ‘post-knowledge test scores compared with pre-knowledge test scores. This change was statistically significant (p=0.011). Upon completion of simulation training on both Mogen and Gomco clamps, all participants (8/8) have chosen to perform circumcision with the Gomco clamp. Both in-person (4/4) and virtual participants (4/4) performed >75% of the simulation and clinical circumcision independently. For self-efficacy, Z score assessment for mean values (changes from baseline) as the effect measure favors post-CIRCLES, except for management of bleeding (**Figure 1** and description in the **appendix**). On a 1-5 Likert scale for overall satisfaction and engagement with both the simulation and clinical practice sessions, all participants rated CIRCLES 5/5. All learners responded that both the simulation and clinical sessions improved their understanding and ability to perform neonatal circumcision on the simulator (5/5). Five learners answered that they felt most engaged during the simulation training and least engaged during the online lecture. When asked what they would do differently in their practices as a result of the course, they answered: “I will be less stressed having done a circumcision on a patient”/”Hopefully will incorporate neonatal circ into future practice”/”Greater ability to counsel on the procedure, risks, suitability, and aftercare, and manage common complications”/ “Considering doing circs.”/”I now know how to circumcise! won’t feel as afraid of circumcisions anymore”/”More confidence in evaluating suitability for circ and knowing when to refer”/”Nothing at this point”/”Ensure proper safety checks and adhering to a step-wise procedure acknowledging things that can go wrong and minimizing risks of complications as much as possible.” Two learners mentioned in person would be more ideal for the simulation.

“However, it works under these circumstances.”

## 4 Discussion

Complications due to neonatal circumcision, although rare, can be devastating and lead to long-term effects on the baby and his parents. These complications can possibly be avoided through formal training and education (4). Despite the recommendation to use local anesthetic during neonatal circumcision, many healthcare providers do not routinely use analgesics during the procedure (7).

A review of pediatric residency training in newborn nurseries in the United States has recommended the provision of instruction on correct circumcision and anesthetic techniques for obstetrics, family medicine, and pediatrics programs (8). Approximately 65% of all male neonates in the US are circumcised (9), and these procedures are performed by healthcare providers with no formal training need to change. A systematic review by Gyan et al. (10) showed that better healthcare provider training and education can lead to a decrease in the short- and long-term morbidity associated with neonatal circumcision. Demaria et al. (4) showed that most physicians performing neonatal circumcision in Southwestern Ontario, Canada, received no formal training. Many of these practitioners are unaware of the contraindications to neonatal circumcision, and most non-surgeons perform the procedure without being able to handle common post-surgical complications. Furthermore, Kim et al. assessed risk factors for surgical complications in neonatal circumcision and identified that a weight above 5.1 kg was a major risk factor (3). For trained practitioners, this can potentially be identified prior to circumcision, and proper preprocedural consulting can be provided to minimize these surgical risks.

Salle et al. reported six cases of penile amputation over a 10 year-period treated at our institution (11). Since then, we have treated 4 more amputation cases, 2 of which occurred in the last year. Sadly, four circumcision-related deaths have been reported in Canada in the last few years. We believe that these events are likely under-reported. The most recent deaths were documented in 2002 (blood loss), 2007 (necrotizing fasciitis), and 2015 (blood loss) (12–14). Non-surgical medical consultants performed the procedure in all cases mentioned above.

Our pilot study showed that pediatric residents and the nurse practitioner, were able to demonstrate significant improvement in their knowledge, skills, and confidence in performing neonatal circumcision after completing the CIRCLES program. This comprehensive program followed a six-step pedagogical framework for procedural skills training: Learn, See, Practice, Prove, Do, and Maintain (15). Participants completed the web-based learning module and attended didactic lectures on day 1 (Learn). They all observed an expert performing circumcision in a clinical setting as well as on the 3D model during the simulation session on day1.

They trained on the 3D model on Day 1 (Practiced) and were assessed at the end of Day 1 (Prove). On day 2, participants came to perform circumcision in the circumcision clinic at a tertiary pediatric hospital under the supervision of a pediatric urologist (Do). All participants were able to perform the procedure independently. Once participants practice and perform neonatal circumcisions, they will likely be able to (Maintain) their skills.

Self-efficacy data showed that the participants were significantly better at screening babies, identifying contraindications to the procedure, bleeding, and dehiscence, upon completion of the training program. Managing bleeding was the only parameter that did not show a significant difference before and after CIRCLES, although this was likely due to the sample size. The small sample size is a weakness of this study; nevertheless, the study provides proof of concept. The other potential criticism of the program is the limited number of in-person training sessions for day one, given COVID-19, leading to a virtual simulation session for some participants. In the assessment of participants from both the virtual and in-person groups, there was no difference in their knowledge or skill demonstration during the day 2 performance in the circumcision clinic. However, two learners mentioned in the experience surveys that in person, the simulation training would be more ideal.

Our data showed that CIRCLES provided the necessary procedural skills to perform neonatal circumcisions in a group of participants with no prior surgical training. We believe that formal procedural training should be a standard practice for healthcare practitioners planning to perform neonatal circumcision, especially because this procedure is often performed by non-pediatric urologists. This program can be potentially useful for pediatricians who take care of these boys after the procedure, given that many providers who perform circumcision do not necessarily see these babies during follow-up. This can help pediatricians identify contraindications to the procedure, post procedural infections, foreskin adhesions, meatal stenosis, and other complications.

The development of this program during the COVID era forced us to use different means and tools to deliver this course in a safe and healthy environment for participants and coaches. Providing this program virtually allowed us to assess and compare the two groups, and the data did not show any significant difference between the in-person and virtual groups, which is interesting. The fact that this program can be provided virtually is a huge advantage and has the potential to provide this program to interested providers worldwide.

## 5 Conclusion

This pilot study demonstrated that CIRCLES is a comprehensive, well-structured program that can be broadly used by healthcare practitioners involved in the care of newborn babies undergoing circumcision. High-quality learning of office-based neonatal circumcision is needed and can potentially decrease the morbidity and mortality associated with this common procedure. The program can also be offered to a wide array of healthcare providers from different specialties and backgrounds, who will be taking care of male neonates worldwide.

## Data availability statement

The original contributions presented in the study are included in the article/**Supplementary Material**. Further inquiries can be directed to the corresponding author.

## Author contributions

JD is the first and corresponding author. She conceptualized the study design, collaborated with acquisition, analysis and interpretation of data, drafted and critically reviewed the manuscript. MK is the senior author. He contributed to the study design, acquisition of data, analysis, and interpretation of data, drafted the manuscript, and revised the manuscript. MM, MC, MP, and AL contributed to the study design, analysis and interpretation of data, and critically reviewed the manuscript. AA collaborated with the study design, acquisition of data, analysis, and interpretation of data, and drafted and revised the manuscript. SV, EL, JK, MR, and AV contributed to the study design, analysis and interpretation of data, and critically reviewed the manuscript. All authors approved the final manuscript as submitted and agreed to be accountable for all aspects of the work. All authors contributed to the article and approved the submitted version.
