# Supplementary figures and images for: CIRCumcision learning experience using simulation: A pilot learning platform for safe neonatal circumcision training offered either virtually or in person
Source: Front Urol. 2023 Jul 7;3:1199194. doi: 10.3389/fruro.2023.1199194 (PMC12327285; doi:10.3389/fruro.2023.1199194)

Appendix: Self-Efficacy Survey for Performing Neonatal Circumcision
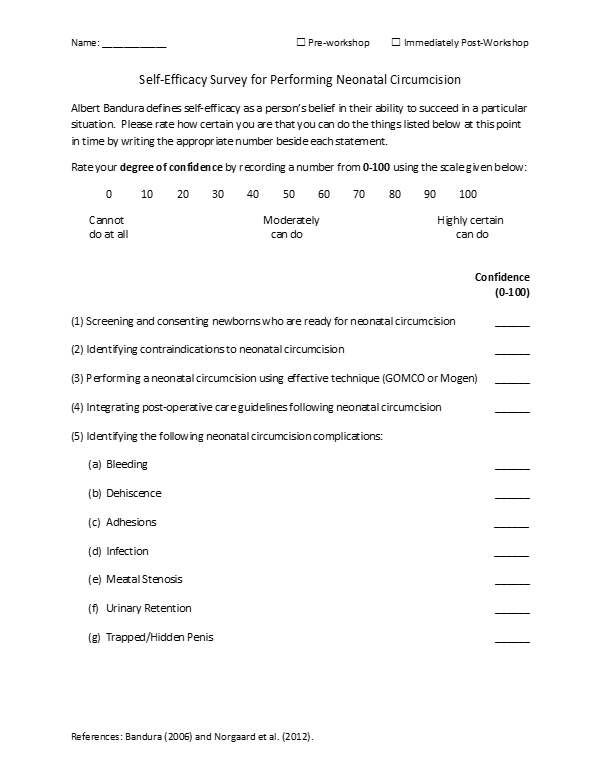

Supplement: Supplementary file 1 [file DataSheet_1.docx]
